# Factors Associated With Alcohol Use Among Individuals Commencing Treatment at Community‐Based Outpatient Treatment Centres in Australia

**DOI:** 10.1111/dar.70034

**Published:** 2025-09-03

**Authors:** Jamie Bryant, Anthony Shakeshaft, Nicholas Lintzeris, Paul Haber, Michael Farrell, Joshua Dizon, Megan Freund

**Affiliations:** ^1^ Health Behaviour Research Collaborative School of Medicine and Public Health, College of Health, Medicine and Wellbeing, University of Newcastle Newcastle Australia; ^2^ Equity in Health and Wellbeing Program Hunter Medical Research Institute Newcastle Australia; ^3^ Poche Centre for Indigenous Health University of Queensland Brisbane Australia; ^4^ Drug and Alcohol Services, South Eastern Sydney Local Health District Sydney Australia; ^5^ Discipline of Addiction Medicine, Faculty of Medicine and Health The University of Sydney Sydney Australia; ^6^ Drug and Alcohol Clinical Research and Improvement Network, NSW Health Sydney Australia; ^7^ Drug Health Services Royal Prince Alfred Hospital Sydney Australia; ^8^ National Drug and Alcohol Research Centre UNSW Sydney Sydney Australia; ^9^ Clinical Research Design and Statistics Hunter Medical Research Institute Newcastle Australia

**Keywords:** alcohol abuse, alcohol consumption, outpatients, socioeconomic factors, treatment

## Abstract

**Introduction:**

Understanding the characteristics of individuals seeking treatment for alcohol use is essential for developing effective interventions. This study aimed to: (i) describe the characteristics of individuals accessing treatment at Australian outpatient alcohol and other drug (AOD) treatment centres; and (ii) identify characteristics associated with more harmful alcohol use at treatment commencement.

**Methods:**

Clients from 34 community‐based AOD centres completed surveys on demographic, substance use, health‐related quality of life and social characteristics. A linear mixed model and two negative binomial models were used to examine factors associated with higher AUDIT scores (indicting more hazardous or harmful alcohol consumption), frequency of drinking days and heavy drinking days in the last 14 days.

**Results:**

Participants (*n* = 1130) were predominantly male (65%), reported concurrent drug use (62%), self‐referred for treatment (57%) and wanted to cease alcohol use completely (42%) or drink moderately (39%). Female gender, unemployment, being a victim or perpetrator of violence, poorer physical and mental health, self‐referral and a goal to cease alcohol use were associated with higher AUDIT scores. Those reporting home duties, retirement, disability/carer pension, student or other employment had lower AUDIT scores. Older age, poorer physical and mental health, and treatment in Western Australia and Queensland were associated with more drinking and heavier drinking days. A goal to cease alcohol use was linked to 23% and 17% lower drinking days and heavy drinking days. Self‐referral was associated with more heavy drinking days.

**Discussion and Conclusion:**

Individuals seeking treatment for AOD use have diverse needs that should inform tailored and holistic treatment.

**Summary**

- Individuals seeking treatment for alcohol misuse at alcohol and other drug (AOD) services are likely to be older, male and smoke tobacco. They are also likely to be experiencing poor mental health and a range of social and economic stressors.
- Higher levels of alcohol use in this treatment‐seeking sample were consistently associated with poorer physical and mental health, and a treatment goal to give up alcohol use completely.
- Clients presenting for AOD treatment have complex needs that may impact their treatment outcomes.
- Promoting the wider uptake of assessment tools specifically designed to measure a range of co‐occurring risk factors may provide AOD services and clinicians with the data on which to develop more integrated and comprehensive models of care for their clients.

## 1 Introduction

Specialist treatment for alcohol use is available from a range of alcohol and other drug (AOD) treatment services. In Australia, these services include a mixture of government, non‐government, and private services that provide outpatient and residential treatments [1, 2] including assessment, withdrawal management, counselling, medications, and case management [1]. In Australia in 2020–21, among people seeking specialist treatment from a government or non‐government AOD treatment service, the most common principal drug of concern was alcohol (42%), for which 87,300 episodes of care were provided [1].

Understanding the characteristics of individuals seeking treatment for alcohol use is critical for tailoring interventions effectively and identifying individuals at higher risk of relapse or poor treatment outcomes. Existing databases like the Australian Alcohol and Other Drug Treatment Services National Minimum Data Set (NMDS) provide an important profile of information about clients who attend specialist AOD services. However, the data are limited as they do not include, for example, information on the level of alcohol dependence, important social factors (e.g., housing risk, health‐related quality of life), important socioeconomic data (e.g., education, employment) or client‐level insights such as the reason for seeking treatment and individual treatment goals, which have been shown to influence treatment engagement, retention, and outcomes [3, 4, 5]. While several studies have explored the characteristics of individuals seeking treatment from AOD for any type of drug use [6, 7, 8], limited research has explored the characteristics of Australian men and women seeking treatment specifically for alcohol dependence nationally with the aim of understanding treatment needs and informing policy. For example, a recent study reported a range of characteristics of clients seeking treatment from public treatment centres for alcohol as their primary drug of concern, but it was limited to only one Australian jurisdiction [9, 10]. In 2016, Lubman and colleagues [11] provided a more comprehensive profile of clients seeking help with alcohol as their primary drug of concern, reporting relatively high rates of social disadvantage, poor quality of life, high severity of substance dependence, and past‐year AOD, mental health, and acute health issues, and social service use. However, this study was limited to only two Australian states, and study participants were more likely to attend withdrawal and long‐term residential care rather than community‐based treatment.

Despite the provision of specialist AOD treatment, such as medically supervised detoxification, residential rehabilitation, and structured counselling, people seeking assistance from specialist AOD treatment services for alcohol dependence often relapse during or after their treatment. Evidence suggests that up to two‐thirds of individuals treated for alcohol use disorder relapse within 6 months [12, 13]. The severity of alcohol dependence has been consistently found to be associated with relapse and poorer treatment outcomes for people who engage in treatment [14]. Recent reviews have reported that in addition to dependence severity, a number of factors are associated with relapse following treatment for alcohol dependence, including psychiatric co‐morbidity, craving, use of other substances, positive family functioning, strong social support, and treatment motivation [15, 16]. A clearer understanding of the profile of clients seeking assistance from community‐based AOD treatment services at a national level can support more effective treatment planning, may reduce the likelihood of client relapse, and assist in developing more targeted and effective interventions across different regions and treatment settings. In addition, understanding levels of dependence and the associated interplay of health and social factors among individuals commencing treatment is essential for developing comprehensive treatment plans that are tailored to the specific needs of individuals with higher levels of alcohol dependence to enhance treatment success.

### 1.1 Aims

This study aimed to: (i) describe the demographic characteristics, drug and alcohol profile, health‐related quality of life, and social circumstances of individuals who seek treatment for alcohol use as their primary substance of concern from Australian outpatient drug and alcohol treatment centres; and (ii) identify the factors associated with more harmful alcohol use at the commencement of treatment.

## 2 Method

### 2.1 Design

This study was nested within a prospective cohort study that was conducted to examine the relative impact of client, treatment, and organisation variables on treatment outcomes for people seeking treatment for alcohol use with alcohol as their primary substance of concern (Australian and New Zealand Clinical Trial Registry ID 371053). Participants completed surveys at the commencement of outpatient alcohol treatment and again 3 months later. Only data pertaining to the baseline survey is reported here. As this paper presents an exploratory analysis of baseline characteristics, a sample size calculation was not applicable in this context.

### 2.2 Treatment Centre Eligibility

Centres were eligible to participate if they provided face‐to‐face specialist outpatient alcohol treatment, with an expected throughput of at least 50 clients reporting alcohol as their primary drug of concern in the next 12 months. Centres that provided primary care, or only provided peer support, residential, or withdrawal services, were excluded.

### 2.3 Treatment Centre Recruitment

Potentially eligible government and non‐government services were identified from the National Drug and Alcohol Services Directory. Potentially eligible services were grouped by their respective state/territory jurisdictions and randomly ordered within each jurisdiction. Proportional random sampling of centres stratified by state/territory was then conducted based on the total number of eligible centres identified in each state/territory. Sequential recruitment of centres was then undertaken with replacement of non‐consenting centres during the recruitment period (December 2016 to May 2018). Centres were contacted by email or telephone by a member of the research team to explore their interest in participating. Interested centres were sent a detailed information statement by the principal investigator to confirm their eligibility and interest, and a teleconference was then organised to discuss participation and receive consent for participation.

### 2.4 Participant Eligibility

Individuals were eligible to participate if they were aged 18 years or older, were presenting for a new episode of outpatient treatment (to ensure the study focused on outcomes of new episodes of treatment and avoided confounding from ongoing treatment) at a participating treatment centre, could read and understand English sufficiently to self‐complete an English language survey, and were judged by treatment centre staff to be mentally and physically well enough to participate. Participants also had to self‐report that alcohol was their primary drug of concern, that they had used alcohol in the last 30 days, that they had commenced treatment in the past 2 weeks, and that they had not previously enrolled in the study in the last 6 months. Participants were excluded if clinic staff judged they were under the influence of drugs or alcohol as they were unable to give informed consent for participation.

### 2.5 Participant Recruitment

Potentially eligible clients were identified by centre staff either through the centre's intake records when the client presented for an initial assessment at the clinic, or at the client's first treatment appointment. Clinic staff provided potentially eligible clients with a verbal overview of the research, detailed written information about the study, and answered any questions. Eligible clients were then invited to complete the survey either before or after their appointment. Participant consent or non‐consent was recorded by clinic staff on a log sheet, and the date of birth and gender of non‐consenting clients was recorded with client permission to allow for determination of non‐consent bias.

### 2.6 Data Collection and Measures

Data were collected between December 2016 and August 2020. Participants completed the survey either on a tablet computer (the majority of participants) or a pen‐ and paper survey if preferred. Participants gave written consent for participation by endorsing a consent statement at the start of the survey. Clients who completed both the baseline and follow‐up survey received a $30 gift voucher (not redeemable for alcohol or tobacco) as reimbursement for their time.

*Demographic characteristics*: Participants self‐reported their age, gender, Aboriginal or Torres Strait Islander status, highest level of completed education, current employment status, main source of income, and current postcode. Participants were also asked to report the number of days they had spent in paid work in the past 4 weeks, and the number of days spent in education or training in the past 4 weeks.

*Alcohol consumption*: Client alcohol consumption was measured using both the 14‐day timeline follow‐back (TLFB) [17] and the alcohol use disorder identification test (AUDIT) [18], both of which are accurate, reliable and valid tools for assessing alcohol consumption and alcohol misuse [19]. The 14‐day TLFB was selected over the more commonly used 28‐day TLFB to increase the accuracy of client recall [20, 21]. To complete the 14‐day TLFB, clients were first asked if they had consumed any alcohol in the last 2 weeks. Those who had not did not complete any additional TLFB questions. Those who had were asked to estimate the number of standard drinks consumed on each of the previous 14 days, with reference to standard drink equivalent information. The AUDIT was used to measure level of risk of harm from alcohol use. Clients completed 10 items, and each item response was given a score ranging from 0 to –4. AUDIT is scored by summing the score of each individual item [18]. Question three of the AUDIT was modified from ‘6 or more drinks’ featured in the original AUDIT to ‘5 or more drinks’ to reflect the Australian guidelines at the time [22].

*Treatment seeking and goal*: Participants were asked author developed questions about who referred them to treatment for their alcohol use (self, family/friends, medical practitioner, hospital, mental health care service, alcohol and other drug treatment service, other community health service, correctional service, police, court, other) and what their treatment goal was (I don't have a clear goal in mind, I would like to give up drinking alcohol completely forever, I would like to give up drinking alcohol at least for a while, I would like to be able to drink moderately or still have a drink occasionally, I don't want to change my drinking at this time, other).

*Other drug use*: Other drug use was measured using questions from the Australian Treatment Outcomes Profile [23]. The Australian Treatment Outcomes Profile is a brief tool that assesses substance use, health and well‐being issues and is increasingly used to monitor clinical outcomes in substance use treatment centres [23]. Clients were asked to indicate whether they had used cannabis, amphetamines, benzodiazepines, heroin, other opioids, cocaine, and tobacco in the past 4 weeks. For each substance selected, clients were asked how many days in each of the past 4 weeks they used that substance.

*Health related quality of life*: Clients completed the Short Form‐8 Health Survey (SF‐8), an 8‐item measure of physical and psychological health and quality of life [24]. The SF‐8 assesses eight dimensions of health including general health perception, physical functioning, role functioning‐physical, bodily pain, vitality, social functioning, mental health, and role functioning‐emotional. Participants respond to each item using a 5‐ or 6‐point Likert scale. Mental and physical sub‐scale scores can be calculated, with higher scores indicating better self‐reported health related quality of life. The SF‐8 is the abbreviated version of the original 36‐item health survey (SF‐36) and has demonstrated reliability and validity across multiple settings [25, 26].

*Social factors*: Social factors were measured using items from the Alcohol Treatment Outcomes Profile instrument [23]. Clients were asked whether in the last 4 weeks they had: been homeless, at risk of being evicted, been arrested, been a victim of violence, or been a perpetrator of violence. Participants were also asked if they were the carer of a child aged 15 years or younger.

### 2.7 Ethics approval

Ethics approval was provided by the University of Newcastle Human Research Ethics Committee (H‐2016‐0385), the Hunter New England Health Research Ethics Committee (HREC; HREC16 HNE193) and relevant state‐based Health Research Ethics Committees.

### 2.8 Statistical Analysis

Descriptive statistics for categorical data are presented as count (%), or mean (SD) and median (min, max) if continuous. Participant postcode was used to determine the level of socioeconomic disadvantage, using the socio‐economic indexes for areas index of relative socio‐economic disadvantage decile classifications [27]. Decile 1 represents the highest level of disadvantage, while decile 10 represents the least disadvantage [27]. Data from the 14 day TLFB was used to derive number of heavy drinking days in the previous 2 weeks, number of drinking days in the previous 2 weeks and total alcohol consumption (number of standard drinks) in the previous 2 weeks [28]. Heavy drinking days were defined as the consumption of five or more standard drinks on any 1 day. This number of standard drinks aligned with Australian guidelines [29]. The AUDIT was scored according to established protocols with a range of possible scores from 0 to 40, with higher scores indicating greater likelihood of hazardous and harmful drinking. Clients scoring one to seven on the AUDIT were classified as at low risk of harmful drinking. Those scoring 8 to 15 were classified as hazardous, 16–19 harmful and 20–40 as high risk [18]. The SF‐8 was also scored according to established protocols. Sub‐scale scores were calculates and then summarised into normalised overall physical health scores and mental health scores [24], with a population mean of 50 and SD of 10 for each measure [24, 30]. The impact of social factors was determined with regard to ‘housing risk’ in last 4 weeks (risk of eviction or experienced homelessness in past 4 weeks), ‘experience of violence’ (perpetrator or victim of violence in the past 4 week), being arrested and being a primary caregiver for child under 15 years of age.

A linear mixed model was used to examine factors associated with the AUDIT total score, and a negative binomial model with a log link was used to examine factors associated with the number of drinking days and number of heavy drinking days in the last 14 days. For all models, fixed effects for gender, age in years, employment status, highest level of education attained, socio‐economic indexes for areas, accessibility/remoteness index, SF‐8 physical component score, SF‐8 mental component score, being at risk of eviction and/or homeless, caring for children, being a victim and/or perpetrator of violence, treatment goal, referral for treatment and state where treatment was being provided, as well as random intercepts for recruitment site. Variables used in modelling were tested for collinearity and no variance inflation factors were found to be > 2.5. Model estimates are given as the mean difference with 95% confidence intervals (CI) for the linear mixed model, and as incident rate ratios (IRR) with 95% CI for the log negative‐binomial models. Due to the large number of omitted observations in the complete‐case models, a sensitivity analysis was performed by pooling regression estimates using Rubin's rules [3] from 30 multiply imputed data sets. Multiple imputation (MI) by fully conditional specification [4, 5, 6, 7] was performed to generate *m* = 30 imputed data sets, under the assumption that the missing data were conditionally missing at random (see Table S2). The imputation model included all independent and outcome variables used in the regression models, as well as additional auxiliary variables identified as predictive of missingness. All analyses were conducted using SAS version 9.4 (SAS Institute Inc., Cary, NC, USA). A priori, *p* < 0.05 (two‐tailed) was used to indicate statistical significance.

## 3 Results

### 3.1 Treatment Centre Sample

Sixty‐five centres across Australia were approached to participate. Of these, five were determined to be ineligible based on the centre inclusion criteria. Of the remaining 60 centres, 36 centres agreed to participate. However, two centres did not commence any participant recruitment and so were excluded from further study participation. A total of 34 centres (60% of those approached and eligible) participated in the study. Participating centres were recruited from New South Wales (*n* = 8), Western Australia (*n* = 7), Queensland (*n* = 5), Victoria (*n* = 5), Tasmania (*n* = 3), the Northern Territory (*n* = 1), Australian Capital Territory (*n* = 2) and South Australia (*n* = 3). Twenty‐one services (62%) were government services, and 13 centres (38%) were non‐government. No private facilities participated in the study. Compared to the national sample of treatment centres [31], centres from Tasmania (2% vs. 9%), Western Australia (10% vs. 21%) and the Australian Capital Territory (1% vs. 6%) were overrepresented in our sample, as were government treatment centres (31% vs. 69%).

### 3.2 Participant Sample and Consent Rate

A total of 1172 participants provided consent and initiated the survey, and 1130 partially or fully completed the TLFBF and/or the AUDIT‐C and were included in the final sample. The raw consent rate was 80% (1172/1463). However, given there was considerable variation in completion of the consent/non‐consent log sheets by treatment centre, a conservative client consent rate was also calculated using the data from centres that were determined to have recorded consent/non‐consent consistently. The criterion for consistency was: regularly recorded clients on log sheets over time (within and over weeks); regularly noted if the client was too unwell to participate; and if pre‐recorded eligible clients on the log sheet based on intake assessment regularly indicated if clients did not attend their appointment. A total of 14 centres met these criteria providing a conservative consent rate of 76%.

Participant demographic characteristics are provided in Table 1. Most of the sample was male (65%) and the mean age of participants was 43 years (standard deviation (SD) = 12). The largest proportion of the sample had a high school education level or below (*n* = 554, 50%), and 54% of participants (*n* = 589) came from areas of high socio‐economic disadvantage (socio‐economic indexes for areas index of relative socio‐economic disadvantage deciles 1–6). Approximately one third of the sample was unemployed (*n* = 369, 33%), while 45% (*n* = 509) were employed either full time or on a part‐time or casual basis. Participants spent an average of 16.3 days in paid work (SD = 7.2) and 8.7 days (SD = 8.1) on education or training in the 4 weeks preceding survey completion.

**TABLE 1:** Participant demographics, *N* = 1130.

Variable	Category	*N*	%
Gender	Male	736	65
	Female	390	35
	Missing	4	—
Currently pregnant (females only)	Yes	3	0.3
	No	387	99.7
	Missing	16	—
Aboriginal or Torres Strait Islander identity	Yes	65	6
	No	1036	94
	Missing	29	—
Highest level of completed education	Secondary school or below	554	50
	Trade or vocational training	353	32
	University degree	200	18
	Missing	23	—
Current employment status	Full‐time, part‐time, or casual work	509	45
	Unemployed	369	33
	Home duties, retired, disability/carer pension, student, or other	243	22
	Missing	9	—
Language other than English spoken at home	Yes	105	10
	No	978	90
	Missing	47	—
Main source of income	Wages and salaries	453	41
	Income from a business	61	5.5
	Property income or superannuation	22	2
	Government pensions or allowances	522	47
	Other	59	5.3
	Missing	13	—
SEIFA	Deciles 1–2	150	14
	Decile 3–4	183	17
	Decile 5–6	265	25
	Decile 7–8	209	19
	Decile 9–10	274	25
	Missing	49	—
Age		43	12 (18–76)
Days in paid work in past 4 weeks		16.3	7.21 (0–28)
Days in education or training in past 4 weeks		8.69	8.14 (0–28)

Abbreviation: SEIFA, socio‐economic indexes for areas.

### 3.3 Alcohol Consumption

In the previous 2 weeks, participants reported an average of 8 days of any drinking (SD = 5, median 9, range 0–14), 7 days of heavy drinking (i.e., consuming five or more standard drinks per day, SD = 5, median = 6, range 0–14), and consumed an average of 84 standard drinks (SD = 78, median = 68, range 0–420). Seventy‐nine percent of the sample met the criteria for high risk of harm based on their AUDIT score, with a further 9.6% classified as harmful drinkers (Table 2).

**TABLE 2:** Drug and alcohol use profile (*n* = 1130).

		N	%
Likelihood of hazardous and harmful drinking (AUDIT overall score)	Low risk	22	2
	Hazardous	103	9.3
	Harmful	106	9.6
	High risk	877	79
	Missing	22	—
Other drug use	None	318	38
	Any	722	65
	Cannabis	237	21
	Amphetamine	79	7.1
	Benzodiazepines	165	15
	Heroin	12	1.1
	Other opioids	40	3.6
	Cocaine	33	3.0
	Tobacco	559	50
	Missing	18	—
Who referred	Self	641	57
	Family/friends	137	12
	Medical practitioner	230	20
	Hospital	97	8.6
	Mental health care service	73	6.5
	Alcohol and other drug treatment service	87	7.7
	Other community health service	28	2.5
	Correctional service/police/court	87	7.7
	Other	28	2.5
	Missing	0	—
Goal for treatment	Give up completely	475	42
	Give up for a while/drink moderately	569	51
	No clear goal/no goal to change drinking habits/other	79	7
	Missing	7	—

Abbreviation: AUDIT, Alcohol Use Disorder Identification Test.

### 3.4 Other Drug Use

Sixty five percent of participants also reported other drug use, mainly tobacco (50%), cannabis (21%) and benzodiazepines (15%) (Table 2).

### 3.5 Referral and Treatment Goal

Many participants self‐referred for treatment (57%) and were seeking to give up alcohol use forever (42%) or be able to drink more moderately (51%) (Table 2).

### 3.6 Quality of Life

SF‐8 normalised sub‐scale scores and component summary scores are provided in Table 3. Participants reported a mean SF‐8 physical component summary score of 45.2 (SD = 10.4) and a mean mental component summary score of 33.9 (SD = 12.1).

**TABLE 3:** Normalised sub‐scale scores and component summary scores for the SF‐8 (*n* = 1130).

Variable	Category	M	SD
Subscale scores	Physical functioning	44.07	9.23
	Role‐physical	42.47	10.00
	Bodily pain	4703	9.96
	General health	39.90	8.46
	Vitality	44.58	8.98
	Social functioning	38.89	10.11
	Role‐ emotional	37.89	9.08
	Mental health	35.42	10.75
Component summary scores	Physical component	45.22	10.44
	Mental component	33.99	12.11

### 3.7 Social Factors

Social factors are provided in Table 4. Almost one quarter of the sample was primary carers of a child aged under 15 years (24%). Fifteen percent of participants had been arrested or been a victim of violence, 14% were at risk of eviction, 12% had been a perpetrator of violence and 11% had experienced homelessness in the past 4 weeks.

**TABLE 4:** Social factors (*n* = 1130).

Social factor		*N*	%
Homeless	Yes	116	11
	No	974	89
	Missing	40	—
At risk of eviction	Yes	156	14
	No	934	86
	Missing	40	—
Eviction risk and/or homeless	Yes	200	18
	No	890	82
	Missing	40	—
Primary carer of child under 15	Yes	259	24
	No	830	76
	Missing	41	—
Been arrested	Yes	164	15
	No	923	85
	Missing	43	—
Been victim of violence	Yes	162	15
	No	925	85
	Missing	43	—
Been perpetrator of violence	Yes	134	12
	No	956	88
	Missing	40	—
Victim and/or perpetrator	Yes	215	20
	No	874	80
	Missing	41	—

### 3.8 Factors Associated With Higher Alcohol Use

*AUDIT total scores*: In the multivariable model, gender, employment status, physical component score and mental component score, being a victim or perpetrator of violence, goal for treatment, and method of referral for treatment were associated with a difference in AUDIT total scores (see Table 5). Being female was associated with a total AUDIT score that was, on average, 0.89 points higher than that of males (mean difference: 0.89; 95% CI 0.08, 1.69; *p* = 0.03). Compared to those in full, part time or casual work, those who reported their employment status as home duties, retired, disability/carer pension, student, or other had a total AUDIT score that was on average 1.47 points lower (mean difference: −1.47, 95% CI −2.51, −0.43; *p* = 0.006), while those who were unemployed had an overall score that was 1.58 points higher (mean difference: 1.58, 95% CI 0.67, 2.49; *p* < 0.001). Each unit increase in the physical component score and the mental component score was associated with a decrease of 0.15 points in overall AUDIT total scores (mean difference: −0.15; 95% CI −0.18, −0.11; *p* < 0.001; and mean difference: −0.16; 95% CI −0.19, −0.13; *p* < 0.001, respectively). Those who had been a victim or perpetrator of violence had on average a 1.05‐point higher total AUDIT score (mean difference: 1.05; 95% CI 0.06, 2.03; *p* < 0.001). Participants whose goal for treatment was to give up completely had on average an AUDIT total score that was 1.44 points higher (mean difference: 1.44, 95% CI 0.65, 2.23; *p* < 0.001) compared to those with other treatment goals. Participants who self‐referred to treatment had an AUDIT score that was 1.70 points higher than those who referred by other means (mean difference: 1.70; 95% CI 0.93, 2.47, *p* < 0.001). In sensitivity analyses (see Table S1), the pooled multivariable regression estimates of factors associated with AUDIT total score were consistent with the corresponding complete‐case model in terms of magnitude and direction of the effects.

**TABLE 5:** Factors associated with higher alcohol use at commencement of treatment, as defined by total AUDIT score, number of drinking days and number of heavy drinking days.

Characteristic	Response	Total AUDIT score				Number of drinking days				Number of heavy drinking days			
		Univariate		Multivariate		Univariate		Multivariate		Univariate		Multivariate	
		Mean difference (95% CI)	*p*	Mean difference (95% CI)	*p*	IRR (95% CI)	*p*	IRR (95% CI)	*p*	IRR (95% CI)	*p*	IRR (95% CI)	*p*
Gender	Male	Reference	—	Reference	—	Reference	—	Reference	—	Reference	—	Reference	—
	Female	1.00 (0.13, 1.86)	0.024	0.89 (0.08, 1.69)	**0.030**	1.04 (0.93, 1.16)	0.470	1.02 (0.91, 1.14)	0.728	1.03 (0.90, 1.18)	0.658	1.00 (0.87, 1.14)	0.983
Age, years		−0.01 (−0.05, 0.03)	0.654	0.01 (−0.03, 0.05)	0.672	1.01 (1.00, 1.01)	0.036	1.01 (1.00, 1.01)	**0.005**	1.01 (1.00, 1.01)	0.061	1.01 (1.00, 1.01)	**0.034**
Employment	Full‐time, part‐time or casual work	Reference	—	Reference	—	Reference	—	Reference	—	Reference	—	Reference	—
	Home duties, retired, disability/carer pension, student, or other	0.06 (−1.00, 1.13)	0.906	−1.47 (−2.51, −0.43)	**0.006**	1.01 (0.88, 1.15)	0.921	0.93 (0.80, 1.07)	0.291	1.03 (0.87, 1.22)	0.701	0.92 (0.77, 1.09)	0.349
	Unemployed	2.99 (2.04, 3.95)	< 0.001	1.58 (0.67, 2.49)	**< 0.001**	0.95 (0.84, 1.07)	0.387	0.92 (0.81, 1.04)	0.189	1.02 (0.88, 1.19)	0.768	0.97 (0.83, 1.13)	0.662
Highest level of education	University degree	Reference	—	Reference	—	Reference	—	Reference	—	Reference	—	Reference	—
	Secondary school or below	0.76 (−0.39, 1.92)	0.195	0.50 (−0.55, 1.55)	0.347	0.96 (0.83, 1.11)	0.589	1.00 (0.87, 1.15)	0.988	0.96 (0.80, 1.14)	0.619	1.00 (0.84, 1.19)	0.997
	Trade or vocational training	0.40 (−0.84, 1.64)	0.530	0.24 (−0.86, 1.34)	0.672	0.95 (0.81, 1.10)	0.477	0.99 (0.85, 1.15)	0.911	0.95 (0.79, 1.15)	0.626	0.98 (0.81, 1.18)	0.819
SEIFA	Deciles 6–10	Reference	—	Reference	—	Reference	—	Reference	—	Reference	—	Reference	—
	Deciles 1–5	0.07 (−0.93, 1.06)	0.895	−0.02 (−0.91, 0.86)	0.961	0.91 (0.81, 1.02)	0.108	0.96 (0.85, 1.108)	0.522	0.88 (0.76, 1.01)	0.075	0.93 (0.80, 1.07)	0.305
ARIA	Major cities of Australia	Reference	—	Reference	—	Reference	—	Reference	—	Reference	—	Reference	—
	Regional or remote Australia	−0.72 (−2.41, 0.97)	0.402	−0.70 (−2.19, 0.79)	0.358	0.85 (0.72, 1.02)	0.075	0.87 (0.73, 1.03)	0.107	0.77 (0.62, 0.96)	0.022	0.83 (0.67, 1.03)	0.093
SF‐8 Physical composite score	—	−0.20 (−0.23, −0.16)	< 0.001	−0.15 (−0.18, −0.11)	**< 0.001**	0.99 (0.99, 1.00)	0.005	0.99 (0.99, 1.00)	**0.003**	0.99 (0.98, 0.99)	< 0.001	0.99 (0.98, 0.99)	**< 0.001**
SF‐8 Mental composite score	—	−0.21 (−0.24, −0.17)	< 0.001	−0.16 (−0.19, −0.13)	**< 0.001**	1.00 (0.99, 1.00)	0.066	1.00 (0.99, 1.00)	**0.037**	0.99 (0.98, 1.00)	< 0.001	0.99 (0.98, 1.00)	**< 0.001**
Eviction risk and/or homeless	No	Reference	—	Reference	—	Reference	—	Reference	—	Reference	—	Reference	—
	Yes	1.17 (0.69, 2.94)	0.001	−0.25 (−1.27, 0.77)	0.629	1.00 (0.87, 1.15)	0.983	1.02 (0.89, 1.19)	0.743	1.01 (0.85, 1.20)	0.907	0.98 (0.82, 1.17)	0.839
Caring for Children	No	Reference	—	Reference	—	Reference	—	Reference	—	Reference	—	Reference	—
	Yes	−1.53 (−2.52, −0.54)	0.003	−0.73 (−1.77, 0.30)	0.164	0.99 (0.88, 1.12)	0.903	1.00 (0.988, 1.13)	0.971	0.96 (0.82, 1.12)	0.629	0.98 (0.83, 1.14)	0.776
Victim and/or perpetrator of violence	No	Reference	—	Reference	—	Reference	—	Reference	—	Reference	—	Reference	—
	Yes	1.94 (0.90, 2.99)	< 0.001	1.05 (0.06, 2.03)	**0.038**	0.96 (0.84, 1.10)	0.529	0.95 (0.83, 1.10)	0.513	0.96 (0.81, 1.14)	0.658	0.95 (0.80, 1.13)	0.556
Treatment goal	Other	Reference	—	Reference	—	Reference	—	Reference	—	Reference	—	Reference	—
	Give up completely	2.72 (1.88–3.57)	< 0.001	1.54 (0.65,2.23)	**0.001**	0.83 (0.75, 0.92)	< 0.001	0.77 (0.69, 0.86)	**< 0.001**	0.92 (0.80, 1.05)	0.197	0.83 (0.72, 0.95)	**0.005**
Referral for treatment	Other	Reference	—	Reference	—	Reference	—	Reference	—	Reference	—	Reference	—
	Self	2.01 (1.16, 2.87)	< 0.001	1.70 (0.93, 2.47)	**< 0.001**	1.11 (1.00, 1.24)	0.047	1.10 (0.99, 1.22)	0.086	1.24 (1.09, 1.42)	0.001	1.21 (1.06, 1.38)	**0.004**
State	New South Wales	Reference	—	Reference	—	Reference	—	Reference	—	Reference	—	Reference	—
	Australian Capital Territory	−4.58 (−9.71, 0.55)	0.080	−2.94 (−6.19, 0.32)	0.077	1.12 (0.81, 1.56)	0.491	1.14 (0.83, 1.58)	0.413	1.12 (0.74, 1.70)	0.598	1.18 (0.80, 1.74)	0.397
	Northern Territory	−7.29 (−15.01, 0.43)	0.064	−3.10 (−8.81, 2.61)	0.287	1.00 (0.51, 1.96)	0.990	1.44 (0.70, 2.93)	0.747	0.89 (0.38, 2.09)	0.792	1.26 (0.54, 2.94)	0.589
	Queensland	−0.30 (−3.49, 2.88)	0.851	−0.59 (−2.59, 1.41)	0.564	1.24 (1.03, 1.50)	0.025	1.36 (1.11, 1.65)	**0.002**	1.30 (1.02, 1.66)	0.032	1.47 (1.17, 1.85)	**0.001**
	South Australia	−1.28 (−5.61, 3.06)	0.563	−1.07 (−3.83, 1.70)	0.451	0.91 (0.67, 1.22)	0.520	0.93 (0.69, 1.25)	0.623	1.03 (0.70, 1.50)	0.896	1.12 (0.78, 1.59)	0.548
	Tasmania	0.24 (−3.90, 4.39)	0.909	0.09 (−2.87, 3.04)	0.954	0.82 (0.63, 1.07)	0.146	0.93 (0.68, 1.27)	0.637	0.81 (0.58, 1.13)	0.215	0.93 (0.64, 1.37)	0.724
	Victoria	−1.29 (−4.76, 2.19)	0.468	−2.04 (−4.24, 0.15)	0.068	1.09 (0.86, 1.37)	0.490	1.11 (0.88, 1.39)	0.375	1.03 (0.77, 1.39)	0.819	1.06 (0.81, 1.39)	0.677
	Western Australia	−1.98 (−5.21, 1.24)	0.228	−1.79 (−3.82, 0.23)	0.083	1.22 (1.00, 1.49)	0.052	1.29 (1.05, 1.58)	**0.014**	1.21 (0.94, 1.56)	0.146	1.30 (1.02, 1.65)	**0.035**

*Note*: Bold values are those that are statistically significant.

Abbreviations: ARIA, Accessibility/Remoteness Index of Australia; AUDIT, Alcohol Use Disorder Identification Test; CI, confidence interval; IRR, incident rate ratios; SEIFA, socio‐economic indexes for areas.

*Number of drinking days*: The multivariable model (see Table 5) includes all available cases (*n* = 777, 69%). In the multivariable model, age, the physical component score and the mental component scores, treatment goal and state where treatment was received were associated with a difference in number of drinking days. Each year increase in age was associated with a 1% increase in number of drinking days (IRR 1.01; 95% CI 1.00, 1.01; *p* = 0.005). Each unit increase in the physical component score was associated with a 1% decrease in number of drinking days respectively (IRR 0.99; 95% CI 0.99, 1.00, *p* = 0.003). Each unit increase in the mental component score was associated with a very small but statistically significant decrease in the number of drinking days (IRR 1.00; 95% CI 0.99, 1.00; *p* = 0.037). Participants who wished to give up alcohol completely had 23% lower number of drinking days (IRR 0.77; 95% CI 0.69, 0.86; *p* < 0.001) compared to those who had other treatment goals. Participants treated in Western Australia had 29% higher number of drinking days (IRR 1.29; 95% CI 1.05, 1.58, *p* = 0.014). In sensitivity analyses (see Table S1), the pooled multivariable regression estimates of factors associated with the number of drinking days were consistent with the corresponding complete‐case model in terms of magnitude and direction of the effects.

*Number of heavy drinking days*: The multivariable model includes all available cases (*n* = 777, 69%). In the multivariable model, age, physical component score, mental component score, treatment goal, source of referral for treatment, and state where treatment was received were significantly associated with a difference in the number of heavy drinking days within the last 14 days. Each year increase in age was associated with a 1% increase in the number of heavy drinking days (IRR 1.01; 95% CI 1.00, 1.01; *p* = 0.034). Each unit increase in physical component score was associated with a 1% lower number of heavy drinking days (IRR 0.99; 95% CI 0.98, 0.99; *p* ≤ 0.001). Similarly, each unit increase in mental component score was associated with a 1% lower number of heavy drinking days (IRR 0.99; 95% CI 0.98, 1.00; *p* < 0.001). Participants who self‐referred for treatment had 21% higher number of heavy drinking days (IRR 1.21; 95% CI 1.06, 1.38; *p* = 0.004) and those who had a treatment goal to give up completely had 17% lower number of heavy drinking days (IRR 0.83; 95% CI 0.672, 0.95; *p* = 0.005). Participants treated in Queensland and Western Australia had 47% and 30% higher number of heavy drinking days (IRR 1.47; 95% CI 1.17, 1.85, *p* = 0.001 and IRR 1.30; 95% CI 1.02, 1.65, *p* = 0.035, respectively). In sensitivity analyses (see Table S1), the pooled multivariable regression estimates of factors associated with the number of heavy drinking days were consistent with the corresponding complete‐case model in terms of magnitude and direction of the effects.

## 4 Discussion

This study provides a comprehensive description of the characteristics of a large sample of clients commencing treatment for alcohol use in community‐based treatment centres across Australia. A wide‐ranging understanding of the complex interplay of factors that affect alcohol use and the success of alcohol treatment is important for informing the design and delivery of treatment programs broadly and tailoring treatment plans to individual needs.

In this study, most clients attending AOD services in Australia seeking treatment for alcohol were male (65%), had a high school or trade level education (76%) and more than a third were unemployed. Participants spent an average of 17 days in paid work and 9 days in study in the month before presenting for treatment. Overall, this client profile, particularly in terms of gender and age, is similar to that of the Australian 2022–23 NMDS for clients receiving treatment for alcohol as the principal drug of concern [32], and recently published data about individuals seeking treatment for alcohol and other drug use in New South Wales (NSW) [10]. In comparison to the NMDS, our sample had fewer Indigenous clients (6% compared to 18%) and was slightly older than the NMDS. The NMDS does not collect information about socioeconomic, educational, or employment status for comparison; however, our national client profile is consistent with NSW‐based treatment‐seeking data in terms of social factors examined, including similar proportions of the samples being homeless or at risk of eviction or arrested in the past 4 weeks. Slightly more of our sample had been a victim or perpetrator of violence in the past 4 weeks (20%) compared to NSW data (11.7%) [10].

The characteristics of the client sample generally reflect those of the Australian population who are more likely to be drinking at risky levels (i.e., who exceed alcohol consumption guidelines). For example, data from the Australian Institute of Health and Welfare show that males are more likely than females to drink at risky levels, as are those aged 50–59 [33]. In addition, those drinking at risky levels were most likely to have completed a trade or vocational training or completed high school (compared to having graduate or post graduate qualification), and were more likely to be employed or unable to work (compared to home duties or being unemployed) [34]. In contrast to our sample, risky drinkers were more likely to come from higher socio‐economic areas [34]. However, the profile of Australians exceeding alcohol consumption guidelines does not necessarily reflect those who need or seek treatment for alcohol use from an AOD service. For example, Proudfoot and colleagues found that while males and those aged 18–34 years were more likely to be alcohol dependent, it was females, those aged between 35 and 54 years (compared to younger and older age groups), and those with higher levels of education, who were more likely to seek help for alcohol [35].

The drug and alcohol profile of participants revealed relatively high levels of alcohol consumption, consuming approximately 84 standard drinks over the 2 week period [22]. Eighty percent of the sample met the criteria for alcohol dependence based on their AUDIT score. Physical component quality of life scores of the sample (mean = 45.2, SD = 10.4) were similar to those reported in other studies of the Australian general population (*n* = 24,872; mean = 47.61, SD = 9.67) [36] and for patients with a chronic illness (*n* = 7606; mean = 42.4, SD = 11.8) [37]. However, mental component quality of life scores of the sample (mean = 34, SD = 12.1) were substantially lower than reported in these previous Australian studies (mean = 50.5, SD = 8.87; [36] and mean = 49.1, SD = 11.1 [37] respectively), indicating substantially poorer psychological health and related quality of life. These results suggest clients seeking treatment for alcohol use continue to consume risky amounts of alcohol and are also experiencing substantial psychological distress or poor mental health. Previous research has similarly suggested that people with a mental health condition are more likely to report drinking alcohol at risky levels [33], and that those with a comorbid affective disorder are more likely to seek treatment for alcohol use [35].

Most participants (62%) in the current sample reported concurrent use of other drugs, with the largest proportion (50%) using tobacco, followed by 21% using cannabis, 15% using benzodiazepines, 7.1% using amphetamines, and 4.7% using opioids in the last month. This level of polydrug use is similar to that found in previous Australian studies conducted with women [7], and in particular with rates of polydrug use reported in a large sample of NSW‐based AOD treatment seekers, which found very similar rates of use of cannabis (19.4%), benzodiazepines (13.7%), amphetamines (5.5%) and opioids (4%) [10]. The NMDS does not report data on concurrent drug use; however, the 2019–20 National Drug Strategy Household Survey showed that around one in five risky drinkers reported recent use of tobacco or cannabis [33]. The source of referrals in the current sample differed slightly from those reported in the NMDS for 2019–20. More clients in the current sample were self‐referred or referred by the criminal justice system compared to the NMDS (57% versus 42% referred by self/family in the NMDS; 7% compared to 2% referred by criminal justice system in the NMDS) [1]. The top treatment goals among clients were complete abstinence (42%), to be able to drink more moderately or occasionally (39%), or to give up drinking alcohol at least for a while (11%). Comparable treatment goals are not reported in the NMDS.

Higher levels of physical and mental health related quality of life were associated with low levels of all three measures of alcohol use (i.e., AUDIT scores and number of drinking and heavy drinking days). This finding supports the well‐established association between alcohol use and poorer physical and mental health [22, 38, 39, 40, 41, 42, 43], and the bi‐directional impact of alcohol consumption and overall health functioning [44, 45]. In addition, self‐referral for treatment was also associated with higher AUDIT total scores and a higher number of heavy drinking days. Although unclear, it may be that the negative impacts on health and decreased social and emotional functioning related to high levels of alcohol consumption prompted self‐referral, indicating higher motivation to change [35, 46, 47]. A 2017 exploratory study found that treatment seeking was associated with the endorsement of more DSM‐IV alcohol dependence criteria and more severe drinking patterns, depression and anxiety, early life stress events and physical neglect, as well as impulsivity and hostility [46]. Participants whose treatment goal was to give up alcohol completely had higher AUDIT total scores (i.e., higher levels of alcohol dependence indicated) but also had a lower number of drinking days and heavy drinking days. Few studies have examined associations between treatment goals and patient characteristics. However, converse to the findings of the current study, a 2017 study in Switzerland found that those who aimed to abstain from alcohol were less likely to report at‐risk alcohol use [3]. Such disparity may be due in part to the differing measures of alcohol risk (AUDIT vs. AUDIT‐C) however, the relationship between client treatment goals and alcohol consumption characteristics warrants further research.

Taken together, the associations identified between alcohol use severity at commencement of treatment and client characteristics highlight important considerations for treatment planning and relapse prevention. Clients presenting with poorer physical and mental health functioning and those who were older had higher alcohol use at treatment commencement on at least two of the three alcohol outcomes examined. Psychiatric co‐morbidity and worse physical health have been linked with higher relapse risk [15], while age has been shown to impact treatment retention among clients receiving outpatient treatment for alcohol misuse [4]. These findings suggest that clients entering treatment with these characteristics may benefit from comprehensive assessment and potentially tailored care plans that address broader psychosocial and health‐related needs. Similarly, the strong association between abstinence‐focused goals and alcohol use at treatment commencement may reflect a group with high motivation but also more severe dependence, reinforcing the need for evidence‐based treatment and relapse prevention strategies. Promoting the wider uptake of assessment tools specifically designed to measure a range of co‐occurring risk factors, such as the ATOP, which has demonstrated reliability and validity in Australian clinical AOD settings [23], may provide AOD services with the data on which to develop integrated and comprehensive models of care for their clients.

### 4.1 Strengths and Limitations

This large national survey included more than 1000 clients seeking treatment for alcohol as their primary drug of concern, and collected novel information about clients' demographics, treatment goals, and social circumstances. However, the study has a number of limitations. Firstly, the timing of survey completion (i.e., before or after a client's intake assessment or first treatment session) varied across participants and was dictated by processes within each participating treatment centre. Client responses may have been influenced by their immediate experiences, expectations, or emotional state at the time of completing the survey, and the recruitment of participants by treatment centre staff may have introduced selection bias, with staff more likely to approach clients who appeared more willing or able to engage, despite eligibility criteria. Clients who consented to complete the survey may have been more motivated to do so, and as such may not be representative of all clients attending the service. These factors are acknowledged as limitations and may have influenced the representativeness and generalisability of the findings. Secondly, all measures in the survey were self‐reported, and as such are subject to potential recall error and social desirability biases. Thirdly, data were collected from a representative sample of 34 out of the more than 1200 publicly funded AOD services nationally [1]. Findings may not be generalisable to the entire national AOD service client base; however, findings are similar to a larger NSW‐based dataset of individuals seeking treatment for alcohol as their primary drug of concern from public AOD services captured through routine comprehensive assessments in the electronic medical record system [10]. We examined the robustness of the results of the complete‐case models to missingness of the data using MI. The pooled regression models from MI yielded results with narrower 95% confidence intervals, but similar effect size estimates, for all effects compared to the respective complete‐case models. Assuming that the missing variables were at random, the sensitivity analysis provides evidence that the complete‐case model results are still likely to be representative of the entire study population despite the high proportion of excluded observations.

## 5 Conclusion

The current findings add to understanding the profile of clients who attend AOD services for alcohol treatment. People seeking treatment for alcohol at AOD services are likely to be older, male and smoke tobacco. They are also likely to be experiencing poor mental health or psychological distress, as well as a range of social and economic stressors. Higher levels of alcohol use in this treatment‐seeking sample were consistently associated with poorer physical and mental health and a goal to give up alcohol completely across the three alcohol outcomes examined. These data support an understanding that clients presenting for AOD treatment often have complex needs that may impact their treatment outcomes.

## Author Contributions

Each author certifies that their contribution to this work meets the standards of the International Committee of Medical Journal Editors.

## Conflicts of Interest

N.L. has received funding from Camurus AB and Indivior for unrelated research and has received honoraria from Camurus AB for presenting at international conference. Constraints on publishing: none. All other authors declare no conflicts of interest.

## Supporting information


**Table S1:** Results of sensitivity analysis showing factors associated with higher alcohol use, as defined by total AUDIT score, number of drinking days, and number of heavy drinking days.
**Table S2:** Participant demographic, drug use, social factors, and SF‐8 scores stratified by missingness of AUDIT total score, Number of drinking days, and Number of heavy drinking days and for the total sample.

## Data Availability

The data that support the findings of this study are available from the corresponding author upon reasonable request.

## References

[1] Australian Institute of Health and Welfare , Alcohol and Other Drug Treatment Services in Australia Annual Report (AIHW, Australian Government, 2021).

[2] A. Ritter , L. Berends , J. Chalmers , P. Hull , K. Lancaster , and M. Gomez , New Horizons: The Review of Alcohol and Other Drug Treatment Services in Australia Final Report (National Drug and Alcohol Research Centre, 2014).

[3] S. Haug , E. Peter , and M. P. Schaub , “Drinking Goals and Their Association With Treatment Retention and Treatment Outcomes Among Clients in Outpatient Alcohol Treatment,” Substance Use & Misuse 52, no. 3 (2017): 313–321. 27767369 10.1080/10826084.2016.1225764

[4] S. Haug and M. P. Schaub , “Treatment Outcome, Treatment Retention, and Their Predictors Among Clients of Five Outpatient Alcohol Treatment Centres in Switzerland,” BMC Public Health 16, no. 1 (2016): 581. 27422382 10.1186/s12889-016-3294-4 PMC4947295

[5] S. J. Adamson , J. D. Sellman , and C. M. A. Frampton , “Patient Predictors of Alcohol Treatment Outcome: A Systematic Review,” Journal of Substance Abuse Treatment 36, no. 1 (2009): 75–86. 18657940 10.1016/j.jsat.2008.05.007

[6] P. A. M. Webster , R. P. Mattick , and A. J. Baillie , “Characteristics of Clients Receiving Treatment in Australian Drug and Alcohol Agencies: A National Census,” Drug and Alcohol Review 11, no. 2 (1992): 111–119. 16840265 10.1080/09595239200185581

[7] W. Swift , J. Copeland , and W. Hall , “Characteristics of Women With Alcohol and Other Drug Problems: Findings of an Australian National Survey,” Addiciton 91, no. 8 (1996): 1141–1150. 10.1046/j.1360-0443.1996.91811416.x 8828242

[8] J. Copeland and W. Hall , “A Comparison of Women Seeking Drug and Alcohol Treatment in a Specialist Women's and Two Traditional Mixed‐Sex Treatment Services,” British Journal of Addiction 87, no. 9 (1992): 1293–1302. 1327337 10.1111/j.1360-0443.1992.tb02738.x

[9] E. Black , R. Bruno , K. Mammen , et al., “Substance Use, Socio‐Demographic Characteristics, and Self‐Rated Health of People Seeking Alcohol and Other Drug Treatment in New South Wales: Baseline Findings From a Cohort Study,” Medical Journal of Australia 219, no. 5 (2023): 218–226. 37449648 10.5694/mja2.52039

[10] N. Heijstee , E. Black , E. Black , et al., “Sociodemographic and Health Factors of the Alcohol Treatment‐Seeking Population in New South Wales, Australia,” Journal of Addiction Medicine 10 (2024): 619–627. 10.1097/ADM.0000000000001311 38828937

[11] D. I. Lubman , J. B. B. Garfield , V. Manning , et al., “Characteristics of Individuals Presenting to Treatment for Primary Alcohol Problems Versus Other Drug Problems in the Australian Patient Pathways Study,” BMC Psychiatry 16, no. 1 (2016): 250. 27435013 10.1186/s12888-016-0956-9 PMC4950603

[12] T. C. Durazzo , MD.“Psychiatric, Demographic, and Brain Morphological Predictors of Relapse After Treatment for an Alcohol Use Disorder,” Alcoholism, Clinical and Experimental Research 41, no. 1 (2017): 107–116. 27883214 10.1111/acer.13267 PMC6193554

[13] S. A. C. Maisto , R. L. Stout , and C. M. Davis , “Drinking in the Year After Treatment as a Predictor of Three‐Year Drinking Outcomes,” Journal of Studies on Alcohol 67, no. 6 (2006): 823–832. 17060998 10.15288/jsa.2006.67.823

[14] M. Kuria , “Factors Associated With Relapse and Remission of Alcohol Dependent Persons After Community Based Treatment,” Open Journal of Psychiatry 3 (2013): 264–272.

[15] W. Sliedrecht , R. de Waart , K. Witkiewitz , and H. G. Roozen , “Alcohol Use Disorder Relapse Factors: A Systematic Review,” Psychiatry Research 278 (2019): 97–115. 31174033 10.1016/j.psychres.2019.05.038

[16] A. B. Yazıcı and M. R. Bardakçı , “Factors Associated With Relapses in Alcohol and Substance Use Disorder,” Eurasian Journal of Medicine 55, no. 1 (2023): S75–S81. 39128053 10.5152/eurasianjmed.2023.23335 PMC11075040

[17] L. C. Sobell and M. B. Sobell , “Timeline Follow‐Back,” in Measuring Alcohol Consumption, ed. R. Z. Litten and J. P. Allen (Springer, 1992), 41–72.

[18] J. B. Saunders , O. G. Aasland , T. F. Babor , J. R. De La Fuente , and M. J. A. Grant , “Development of the Alcohol Use Disorders Identification Test (AUDIT): WHO Collaborative Project on Early Detection of Persons With Harmful Alcohol Consumption‐II,” Addiction 88, no. 6 (1993): 791–804. 8329970 10.1111/j.1360-0443.1993.tb02093.x

[19] R. D. Hays , J. F. Merz , and R. Nicholas , “Response Burden, Reliability, and Validity of the CAGE, Short MAST, and AUDIT Alcohol Screening Measures,” Behavior Research Methods, Instruments, & Computers 27, no. 2 (1995): 277–280.

[20] P. L. Dulin , C. E. Alvarado , J. M. Fitterling , and V. M. Gonzalez , “Comparisons of Alcohol Consumption by Timeline Follow Back vs. Smartphone‐Based Daily Interviews,” Addiction Research and Theory 25, no. 3 (2017): 195–200. 29170622 10.1080/16066359.2016.1239081 PMC5695707

[21] B. B. Hoeppner , R. L. Stout , K. M. Jackson , and N. P. Barnett , “How Good Is Fine‐Grained Timeline Follow‐Back Data? Comparing 30‐Day TLFB and Repeated 7‐Day TLFB Alcohol Consumption Reports on the Person and Daily Level,” Addictive Behaviors 35, no. 12 (2010): 1138–1143. 20822852 10.1016/j.addbeh.2010.08.013 PMC2942970

[22] National Health and Medical Reserach Council , Australian Guidelines to Reduce Health Risks From Drinking Alcohol (NHMRC, 2020).

[23] N. Lintzeris , L. A. Monds , G. Rivas , S. Leung , A. Withall , and B. Draper , “The Australian Treatment Outcomes Profile Instrument as a Clinical Tool for Older Alcohol and Other Drug Clients: A Validation Study,” Drug and Alcohol Review 35, no. 6 (2016): 673–677. 27072851 10.1111/dar.12393

[24] J. Ware , M. Kosinski , J. Dewey , and B. Gandek , How to Score and Interpret Single‐Item Health Status Measures: A Manual for Users of the SF‐8 Health Survey (QualityMetric Incorporated, 2001).

[25] J. Vallès , M. Guilera , Z. Briones , et al., “Validity of the Spanish 8‐Item Short‐Form Generic Health‐Related Quality‐Of‐Life Questionnaire in Surgical Patients: A Population‐Based Study,” Anesthesiology 112, no. 5 (2010): 1164–1174. 20418697 10.1097/ALN.0b013e3181d3e017

[26] B. Roberts , J. Browne , K. F. Ocaka , T. Oyok , and E. Sondorp , “The Reliability and Validity of the SF‐8 With a Conflict‐Affected Population in Northern Uganda,” Health and Quality of Life Outcomes 6, no. 1 (2008): 108. 19055716 10.1186/1477-7525-6-108 PMC2612648

[27] Australian Bureau of Statistics , Information Paper: An Introduction to Socio‐Economic Indexes for Areas (SEIFA), 2006. Cat no. 2039.0 (ABS, 2017).

[28] L. C. Sobell , S. Agrawal , M. B. Sobell , et al., “Comparison of a Quick Drinking Screen With the Timeline Followback for Individuals With Alcohol Problems,” Journal of Studies on Alcohol 64, no. 6 (2003): 858–861. 14743950 10.15288/jsa.2003.64.858

[29] K. A. Brussen , “The Australian Guidelines to Reduce Health Risks From Drinking Alcohol,” 15, no. 3 (2010): 9–12.

[30] C. Taft , J. Karlsson , and M. Sullivan , “Do SF‐36 Summary Component Scores Accurately Summarize Subscale Scores?,” Quality of Life Research 10, no. 5 (2001): 395–404. 11763202 10.1023/a:1012552211996

[31] Australian Institute of Health and Welfare , “Alcohol and Other Drug Treatment Services in Australia Annual Report,” 2024.

[32] Australian Institute of Health and Welfare , Alcohol and Other Drug Treatment Services National Minimum Data Set (AODTS NMDS) (Australian Government, 2022).

[33] Australian Institute of Health and Welfare , Alcohol, Tobacco & Other Drugs in Australia (AIHW, 2021), https://www.aihw.gov.au/reports/alcohol/alcohol‐tobacco‐other‐drugs‐australia/contents/drug‐types/alcohol.

[34] Australian Institute of Health and Welfare , Alcohol Risk and Harm (AIHW, 2021), https://www.aihw.gov.au/reports/australias‐health/alcohol‐risk‐and‐harm.

[35] H. Proudfoot and M. Teesson , “Who Seeks Treatment for Alcohol Dependence?,” Social Psychiatry and Psychiatric Epidemiology 37, no. 10 (2002): 451–456. 12242622 10.1007/s00127-002-0576-1

[36] M. R. Le Grande , G. Tucker , S. Bunker , and A. C. Jackson , “Validating the Short Form‐12 and the Development of Disease‐Specific Norms in a Cohort of Australian Private Health Insurance Members,” Australian Journal of Primary Health 25, no. 1 (2019): 90–96. 30711020 10.1071/PY18069

[37] U. W. Jayasinghe , J. Proudfoot , C. A. Barton , et al., “Quality of Life of Australian Chronically‐Ill Adults: Patient and Practice Characteristics Matter,” Health and Quality of Life Outcomes 7 (2009): 50. 19493336 10.1186/1477-7525-7-50 PMC2700088

[38] K. U. Gomez , O. McBride , E. Roberts , et al., “The Clustering of Physical Health Conditions and Associations With Co‐Occurring Mental Health Problems and Problematic Alcohol Use: A Cross‐Sectional Study,” BMC Psychiatry 23, no. 1 (2023): 89. 36747152 10.1186/s12888-023-04577-3 PMC9901006

[39] E. Roberts , R. Morse , S. Epstein , M. Hotopf , D. Leon , and C. Drummond , “The Prevalence of Wholly Attributable Alcohol Conditions in the United Kingdom Hospital System: A Systematic Review, Meta‐Analysis and Meta‐Regression,” Addiction 114, no. 10 (2019): 1726–1737. 31269539 10.1111/add.14642 PMC6771834

[40] G. Danaei , S. Vander Hoorn , A. D. Lopez , C. J. Murray , and M. Ezzati , “Causes of Cancer in the World: Comparative Risk Assessment of Nine Behavioural and Environmental Risk Factors,” Lancet 366, no. 9499 (2005): 1784–1793. 16298215 10.1016/S0140-6736(05)67725-2

[41] D. O. Baliunas , B. J. Taylor , H. Irving , et al., “Alcohol as a Risk Factor for Type 2 Diabetes: A Systematic Review and Meta‐Analysis,” Diabetes Care 32, no. 11 (2009): 2123–2132. 19875607 10.2337/dc09-0227 PMC2768203

[42] S. S. Lim , T. Vos , A. D. Flaxman , et al., “A Comparative Risk Assessment of Burden of Disease and Injury Attributable to 67 Risk Factors and Risk Factor Clusters in 21 Regions, 1990‐2010: A Systematic Analysis for the Global Burden of Disease Study 2010,” Lancet 380, no. 9859 (2012): 2224–2260. 23245609 10.1016/S0140-6736(12)61766-8 PMC4156511

[43] E. F. Mathiesen , S. Nome , M. Eisemann , and J. Richter , “Drinking Patterns, Psychological Distress and Quality of Life in a Norwegian General Population‐Based Sample,” Quality of Life Research 21, no. 9 (2012): 1527–1536. 22219172 10.1007/s11136-011-0080-8

[44] A. Salonsalmi , O. Rahkonen , E. Lahelma , and M. Laaksonen , “The Association Between Alcohol Drinking and Self‐Reported Mental and Physical Functioning: A Prospective Cohort Study Among City of Helsinki Employees,” BMJ Open 7, no. 4 (2017): e014368. 10.1136/bmjopen-2016-014368 PMC5623368 28473511

[45] J. D. Swendsen and K. R. Merikangas , “The Comorbidity of Depression and Substance Use Disorders,” Clinical Psychology Review 20, no. 2 (2000): 173–189. 10721496 10.1016/s0272-7358(99)00026-4

[46] M. C. H. Rohn , M. R. Lee , S. B. Kleuter , M. L. Schwandt , D. E. Falk , and L. Leggio , “Differences Between Treatment‐Seeking and Nontreatment‐Seeking Alcohol‐Dependent Research Participants: An Exploratory Analysis,” Alcoholism, Clinical and Experimental Research 41, no. 2 (2017): 414–420. 28129451 10.1111/acer.13304 PMC6468994

[47] P. C. D'Souza and P. J. Mathai , “Motivation to Change and Factors Influencing Motivation in Alcohol Dependence Syndrome in a Tertiary Care Hospital,” Indian Journal of Psychiatry 59, no. 2 (2017): 183–188. 28827865 10.4103/psychiatry.IndianJPsychiatry_262_15 PMC5547859

