# Supplementary material for: Factors Associated With Alcohol Use Among Individuals Commencing Treatment at Community‐Based Outpatient Treatment Centres in Australia
Source: Drug Alcohol Rev. 2025 Sep 3;44(7):2066–79. doi: 10.1111/dar.70034 (PMC12581934; doi:10.1111/dar.70034)
Supplement: Supplementary file 1 — Table S1: Results of sensitivity analysis showing factors associated with higher alcohol use, as defined by total AUDIT score, number of drinking days, and number of heavy drinking days. Table S2: Participant demographic, drug use, social factors, and SF‐8 scores stratified by missingness of AUDIT total score, Number of drinking days, and Number of heavy drinking days and for the total sample. [file DAR-44-2066-s001.docx]

**Table S1. Results of sensitivity analysis showing factors associated with higher alcohol use, as defined by total AUDIT score, number of drinking days, and number of heavy drinking days.**

|  |  | **Total AUDIT score** | | **Number of drinking days** | | **Number of heavy drinking days** | | |
| --- | --- | --- | --- | --- | --- | --- | --- | --- |
| **Characteristic** | **Response** | **Mean difference (95% CI)** | ***p*-value** | **IRR (95%CI)** | ***p*-value** | **IRR (95% CI)** | | ***p*-value** |
| Gender | Male | Reference | - | Reference | - | Reference | - | |
|  | Female | 0.89 (0.09, 1.69) | **0.030** | 1.02 (0.91, 1.14) | 0.711 | 1.00 (0.87, 1.14) | 0.994 | |
| Age, years |  | 0.00 (-0.03, 0.04) | 0.816 | 1.01 (1.00, 1.01) | **0.009** | 1.01 (1.00, 1.01) | **0.041** | |
| Employment | Full-time, part-time, or casual work | Reference | - | Reference | - | Reference | - | |
|  | Home duties, retired, disability/carer pension, student or other | -1.47 (-2.52, -0.43) | **0.006** | 0.93 (0.81, 1.08) | 0.336 | 0.93 (0.78, 1.11) | 0.439 | |
|  | Unemployed | 1.56 (0.65, 2.48) | **<0.001** | 0.92 (0.81, 1.04) | 0.182 | 0.97 (0.83, 1.13) | 0.660 | |
| Highest level of education | University degree | Reference | - | Reference | - | Reference | - | |
|  | Secondary school or below | 0.49 (-0.55, 1.53) | 0.358 | 1.01 (0.87, 1.17) | 0.907 | 1.00 (0.84, 1.20) | 0.962 | |
|  | Trade or vocational training | 0.25 (-0.85, 1.35) | 0.658 | 1.00 (0.86, 1.16) | 0.970 | 0.99 (0.82, 1.19) | 0.911 | |
| SEIFA | Deciles 6-10 | Reference | - | Reference | - | Reference | - | |
|  | Deciles 1-5 | -0.00 (-0.88, 0.88) | 0.996 | 0.95 (0.84, 1.07) | 0.403 | 0.92 (0.79, 1.06) | 0.249 | |
| ARIA | Major cities of Australia | Reference | - | Reference | - | Reference | - | |
|  | Regional or remote Australia | -0.73 (-2.22, 0.76) | 0.339 | 0.87 (0.73, 1.04) | 0.117 | 0.83 (0.67, 1.03) | 0.094 | |
| Physical QoL | - | -0.15 (-0.19, -0.11) | **<0.001** | 0.99 (0.99, 1.00) | **0.004** | 0.99 (0.98, 0.99) | **<0.001** | |
| Mental QoL | - | -0.16 (-0.20, -0.13) | **<0.001** | 1.00 (0.99, 1.00) | **0.047** | 0.99 (0.98, 1.00) | **0.001** | |
| Eviction risk and/or homeless | No | Reference | - | Reference | - | Reference | - | |
|  | Yes | -0.26 (-1.28, 0.76) | 0.613 | 1.04 (0.90, 1.20) | 0.585 | 1.00 (0.83, 1.19) | 0.959 | |
| Caring for Children | No | Reference | - | Reference | - | Reference | - | |
|  | Yes | -0.89 (-1.80, 0.02) | 0.055 | 0.99 (0.88, 1.13) | 0.935 | 0.98 (0.84, 1.15) | 0.818 | |
| Victim and/or perpetrator of violence | No | Reference | - | Reference | - | Reference | - | |
|  | Yes | 1.06 (0.07, 2.04) | 0.035 | 0.95 (0.82, 1.09) | 0.458 | 0.95 (0.79, 1.13) | 0.534 | |
| Treatment goal | Other | Reference | - | Reference | - | Reference | - | |
|  | Give up completely | 1.43 (0.64, 2.22) | **<0.001** | 0.78 (0.70, 0.87) | **<0.001** | 0.83 (0.73, 0.95) | **0.008** | |
| Referral for treatment | Other | Reference | - | Reference | - | Reference | - | |
|  | Self | 1.70 (0.93, 2.47) | **<0.001** | 1.10 (0.99, 1.22) | 0.090 | 1.21 (1.06, 1.38) | **0.005** | |
| State | NSW | Reference | - | Reference | - | Reference | - | |
|  | Australian Capital Territory | -2.95 (-6.20, 0.31) | 0.076 | 1.14 (0.83, 1.55) | 0.423 | 1.18 (0.81, 1.72) | 0.392 | |
|  | Northern Territory | -3.08 (-8.79, 2.63) | 0.291 | 1.39 (0.68, 2.81) | 0.367 | 1.24 (0.54, 2.88) | 0.614 | |
|  | Queensland | -0.60 (-2.60, 1.41) | 0.559 | 1.35 (1.13, 1.63) | **0.001** | 1.47 (1.17, 1.84) | **<0.001** | |
|  | South Australia | -1.08 (-3.86, 1.69) | 0.444 | 0.94 (0.70, 1.26) | 0.683 | 1.12 (0.79, 1.58) | 0.520 | |
|  | Tasmania | 0.10 (-2.86, 3.05) | 0.949 | 0.92 (0.68, 1.26) | 0.607 | 0.94 (0.64, 1.37) | 0.730 | |
|  | Victoria | -2.05 (-4.25, 0.15) | 0.068 | 1.11 (0.89, 1.39) | 0.364 | 1.06 (0.81, 1.38) | 0.693 | |
|  | Western Australia | -1.80 (-3.83, 0.23) | 0.082 | 1.28 (1.05, 1.55) | **0.013** | 1.28 (1.01, 1.63) | **0.038** | |

ARIA, Accessibility/Remoteness Index of Australia; AUDIT, Alcohol Use Disorder Identification Test; CI, confidence interval; IRR, incident rate ratios; QoL, quality of life; SEIFA, Socio-Economic Indexes for Areas.

**Table S2:** Participant demographic, drug use, social factors, and SF-8 scores stratified by missingness of AUDIT total score, Number of drinking days, and Number of heavy drinking days and for the total sample.

|  | | **Total audit score** | | | | **Number of drinking days** | | | **Number of heavy drinking days** | | | **Total sample** | |
| --- | --- | --- | --- | --- | --- | --- | --- | --- | --- | --- | --- | --- | --- |
| **Demographics** | **Response** | **Non-missing**  **(n=1108)** | **Missing**  **(n=22)** | ***p*-value** | **Non-missing (n=1030)** | | **Missing (n=100)** | ***p*-value** | **Non-missing (n=1030)** | **Missing (n=100)** | ***p*-value** | **Total**  **(N=1130)** |  |
| **Gender** | Male | 721 (65%) | 15 (79%) | 0.2095 | 661 (64%) | | 75 (76%) | **0.0229** | 661 (64%) | 75 (76%) | **0.0229** | 736 (65%) |  |
|  | Female | 386 (35%) | 4 (21%) |  | 366 (36%) | | 24 (24%) |  | 366 (36%) | 24 (24%) |  | 390 (35%) |  |
|  | *Missing* | 1 | 3 |  | 3 | | 1 |  | 3 | 1 |  | 4 |  |
| **Currently pregnant** | Yes | 3 (0.3%) | - | 0.4842 | 3 (0.3%) | | - | 0.0953 | 3 (0.3%) |  | 0.0953 | 3 (0.3%) |  |
|  | *Missing* | 13 | 3 |  | 15 | | 1 |  | 15 | 1 |  | 16 |  |
| **Indigenous**  **status** | No | 1018 (94%) | 18 (95%) | 0.9049 | 946 (94%) | | 90 (91%) | 0.1584 | 946 (94%) | 90 (91%) | 0.1584 | 1036 (94%) |  |
|  | Yes, Aboriginal and/or Torres Strait Islander | 64 (5.9%) | 1 (5.3%) |  | 56 (5.6%) | | 9 (9.1%) |  | 56 (5.6%) | 9 (9.1%) |  | 65 (5.9%) |  |
|  | *Missing* | 26 | 3 |  | 28 | | 1 |  | 28 | 1 |  | 29 |  |
| **Education** | Secondary school or below | 543 (50%) | 11 (61%) | 0.3768 | 499 (49%) | | 55 (56%) | 0.2450 | 499 (49%) | 55 (56%) | 0.2450 | 554 (50%) |  |
|  | Trade or vocational training | 350 (32%) | 3 (17%) |  | 322 (32%) | | 31 (32%) |  | 322 (32%) | 31 (32%) |  | 353 (32%) |  |
|  | University degree | 196 (18%) | 4 (22%) |  | 188 (19%) | | 12 (12%) |  | 188 (19%) | 12 (12%) |  | 200 (18%) |  |
|  | *Missing* | 19 | 4 |  | 21 | | 2 |  | 21 | 2 |  | 23 |  |
| **Employment**  **status** | Full-time, part-time, casual work | 499 (45%) | 10 (56%) | 0.6792 | 479 (47%) | | 30 (31%) | **0.0059** | 479 (47%) | 30 (31%) | **0.0059** | 509 (45%) |  |
|  | Unemployed | 364 (33%) | 5 (28%) |  | 325 (32%) | | 44 (45%) |  | 325 (32%) | 44 (45%) |  | 369 (33%) |  |
|  | Home duties, retired, disability/ carer pension, student | 240 (22%) | 3 (17%) |  | 219 (21%) | | 24 (24%) |  | 219 (21%) | 24 (24%) |  | 243 (22%) |  |
|  | *Missing* | 5 | 4 |  | 7 | | 2 |  | 7 | 2 |  | 9 |  |
| **Speak language other than English at home** | No | 962 (90%) | 16 (94%) | 0.5923 | 895 (90%) | | 83 (89%) | 0.7185 | 895 (90%) | 83 (89%) | 0.7185 | 978 (90%) |  |
|  | Yes | 104 (9.8%) | 1 (5.9%) |  | 95 (9.6%) | | 10 (11%) |  | 95 (9.6%) | 10 (11%) |  | 105 (9.7%) |  |
|  | *Missing* | 42 | 5 |  | 40 | | 7 |  | 40 | 7 |  | 47 |  |
| **Main income source** | Wages and salaries | 444 (40%) | 9 (50%) | 0.9088 | 429 (42%) | | 24 (25%) | **0.0106** | 429 (42%) | 24 (25%) | **0.0106** | 453 (41%) |  |
|  | Income from a business | 60 (5.5%) | 1 (5.6%) |  | 56 (5.5%) | | 5 (5.2%) |  | 56 (5.5%) | 5 (5.2%) |  | 61 (5.5%) |  |
|  | Property income or superannuation | 22 (2.0%) | - |  | 21 (2.1%) | | 1 (1.0%) |  | 21 (2.1%) | 1 (1.0%) |  | 22 (2.0%) |  |
|  | Government pensions or allowances | 515 (47%) | 7 (39%) |  | 461 (45%) | | 61 (63%) |  | 461 (45%) | 61 (63%) |  | 522 (47%) |  |
|  | Other | 58 (5.3%) | 1 (5.6%) |  | 53 (5.2%) | | 6 (6.2%) |  | 53 (5.2%) | 6 (6.2%) |  | 59 (5.3%) |  |
|  | *Missing* | 9 | 4 |  | 10 | | 3 |  | 10 | 3 |  | 13 |  |
| **SEIFA** | Deciles 1-2 | 147 (14%) | 3 (17%) | 0.6768 | 135 (14%) | | 15 (17%) | 0.5063 | 135 (14%) | 15 (17%) | 0.5063 | 150 (14%) |  |
|  | Deciles 3-4 | 180 (17%) | 3 (17%) |  | 168 (17%) | | 15 (17%) |  | 168 (17%) | 15 (17%) |  | 183 (17%) |  |
|  | Deciles 5-6 | 260 (24%) | 5 (28%) |  | 239 (24%) | | 26 (30%) |  | 239 (24%) | 26 (30%) |  | 265 (25%) |  |
|  | Deciles 7-8 | 204 (19%) | 5 (28%) |  | 194 (20%) | | 15 (17%) |  | 194 (20%) | 15 (17%) |  | 209 (19%) |  |
|  | Deciles 9-10 | 272 (26%) | 2 (11%) |  | 257 (26%) | | 17 (19%) |  | 257 (26%) | 17 (19%) |  | 274 (25%) |  |
|  | Deciles 1-5 | 485 (46%) | 7 (39%) | 0.5693 | 448 (45%) | | 44 (50%) | 0.3779 | 448 (45%) | 44 (50%) | 0.3779 | 492 (46%) |  |
|  | Deciles 6-10 | 578 (54%) | 11 (61%) |  | 545 (55%) | | 44 (50%) |  | 545 (55%) | 44 (50%) |  | 589 (54%) |  |
|  | *Missing* | 45 | 4 |  | 37 | | 12 |  | 37 | 12 |  | 49 |  |
| **ARIA** | Major cities of Australia | 777 (73%) | 13 (72%) | 0.9393 | 731 (74%) | | 59 (66%) | 0.1360 | 731 (74%) | 59 (66%) | 0.1360 | 790 (73%) |  |
|  | Regional or remote Australia | 287 (27%) | 5 (28%) |  | 262 (26%) | | 30 (34%) |  | 262 (26%) | 30 (34%) |  | 292 (27%) |  |
|  | *Missing* | 44 | 4 |  | 37 | | 11 |  | 37 | 11 |  | 48 |  |
| **Age, years** | mean (SD) | 43 (12) | 40 (12) | 0.3197 | 43 (12) | | 46 (11) | **0.0450** | 43 (12) | 46 (11) | **0.0450** | 43 (12) |  |
|  | median (min, max) | 43 (18, 76) | 44 (24, 58) | 0.3880 | 43 (18, 76) | | 46 (22, 71) | **0.0398** | 43 (18, 76) | 46 (22, 71) | **0.0398** | 43 (18, 76) |  |
| **Days in paid work in last 4 weeks** | mean (SD) | 16.28 (7.22) | 16.83 (7.25) | 0.7937 | 16.29 (7.18) | | 16.41 (7.80) | 0.9338 | 16.29 (7.18) | 16.41 (7.80) | 0.9338 | 16.30 (7.21) |  |
|  | median (min, max) | 20.00 (0.00, 28.00) | 20.00 (1.00, 24.00) | 0.6005 | 20.00 (0.00, 28.00) | | 20.00 (1.00, 28.00) | 0.6742 | 20.00 (0.00, 28.00) | 20.00 (1.00, 28.00) | 0.6742 | 20.00 (0.00, 28.00) |  |
| **Days in education or training in last 4 weeks** | mean (SD) | 8.80 (8.18) | 3.00 (.) | 0.4856 | 8.04 (7.67) | | 15.20 (10.76) | 0.0600 | 8.04 (7.67) | 15.20 (10.76) | 0.0600 | 8.69 (8.14) |  |
|  | median (min, max) | 6.00 (0.00, 28.00) | 3.00 (3.00, 3.00) | 0.4482 | 6.00 (0.00, 28.00) | | 20.00 (3.00, 28.00) | 0.1419 | 6.00 (0.00, 28.00) | 20.00 (3.00, 28.00) | 0.1419 | 6.00 (0.00, 28.00) |  |
| **Drug use** | Cannabis | 237 (21%) | - | 0.2012 | 218 (21%) | | 19 (22%) | 0.9471 | 218 (21%) | 19 (22%) | 0.9471 | 237 (21%) |  |
|  | Amphetamine | 79 (7.1%) | - | 0.4970 | 67 (6.5%) | | 12 (14%) | **0.0129** | 67 (6.5%) | 12 (14%) | **0.0129** | 79 (7.1%) |  |
|  | Benzodiazepines | 165 (15%) | - | 0.3053 | 151 (15%) | | 14 (16%) | 0.7684 | 151 (15%) | 14 (16%) | 0.7684 | 165 (15%) |  |
|  | Heroin | 12 (1.1%) | - | 0.7975 | 9 (0.9%) | | 3 (3.4%) | **0.0275** | 9 (0.9%) | 3 (3.4%) | **0.0275** | 12 (1.1%) |  |
|  | Opioids | 40 (3.6%) | - | 0.6352 | 36 (3.5%) | | 4 (4.5%) | 0.6186 | 36 (3.5%) | 4 (4.5%) | 0.6186 | 40 (3.6%) |  |
|  | Cocaine | 33 (3.0%) | - | 0.6675 | 31 (3.0%) | | 2 (2.3%) | 0.6889 | 31 (3.0%) | 2 (2.3%) | 0.6889 | 33 (3.0%) |  |
|  | Tobacco | 558 (50%) | 1 (17%) | 0.0988 | 512 (50%) | | 47 (53%) | 0.5394 | 512 (50%) | 47 (53%) | 0.5394 | 559 (50%) |  |
|  | None of the above | 314 (28%) | 4 (67%) | **0.0385** | 300 (29%) | | 18 (20%) | 0.0782 | 300 (29%) | 18 (20%) | 0.0782 | 318 (29%) |  |
|  | *Missing* | 2 | 16 | - | 6 | | 12 | - | 6 | 12 | - | 18 |  |
|  | Any | 721 (65%) | 1 (17%) | **0.0130** | 660 (64%) | | 62 (70%) | 0.2576 | 660 (64%) | 62 (70%) | 0.2576 | 722 (65%) |  |
|  | Any (excluding tobacco) | 392 (35%) | - | 0.0699 | 358 (35%) | | 34 (39%) | 0.4886 | 358 (35%) | 34 (39%) | 0.4886 | 392 (35%) |  |
| **Homeless** | No | 973 (89%) | 1 (50%) | 0.0708 | 907 (90%) | | 67 (80%) | **0.0030** | 907 (90%) | 67 (80%) | **0.0030** | 974 (89%) |  |
|  | Missing | 20 | 20 |  | 24 | | 16 |  | 24 | 16 |  | 40 |  |
| **At risk of eviction** | Yes | 155 (14%) | 1 (50%) | 0.1492 | 131 (13%) | | 25 (30%) | **<0.0001** | 131 (13%) | 25 (30%) | **<0.0001** | 156 (14%) |  |
|  | No | 933 (86%) | 1 (50%) |  | 875 (87%) | | 59 (70%) |  | 875 (87%) | 59 (70%) |  | 934 (86%) |  |
|  | Missing | 20 | 20 |  | 24 | | 16 |  | 24 | 16 |  | 40 |  |
| **Eviction risk and/or homeless** | Yes | 199 (18%) | 1 (50%) | 0.2471 | 168 (17%) | | 32 (38%) | **<0.0001** | 168 (17%) | 32 (38%) | **<0.0001** | 200 (18%) |  |
|  | No | 889 (82%) | 1 (50%) |  | 838 (83%) | | 52 (62%) |  | 838 (83%) | 52 (62%) |  | 890 (82%) |  |
|  | Missing | 20 | 20 |  | 24 | | 16 |  | 24 | 16 |  | 40 |  |
| **Primary carer of child under 15** | Yes | 259 (24%) | - | 0.4291 | 246 (24%) | | 13 (15%) | **0.0627** | 246 (24%) | 13 (15%) | **0.0627** | 259 (24%) |  |
|  | No | 828 (76%) | 2 (100.0%) |  | 759 (76%) | | 71 (85%) |  | 759 (76%) | 71 (85%) |  | 830 (76%) |  |
|  | Missing | 21 | 20 |  | 25 | | 16 |  | 25 | 16 |  | 41 |  |
| **Been arrested** | Yes | 162 (15%) | 2 (100.0%) | **0.0008** | 135 (13%) | | 29 (35%) | **<0.0001** | 135 (13%) | 29 (35%) | **<0.0001** | 164 (15%) |  |
|  | No | 923 (85%) | - |  | 868 (87%) | | 55 (65%) |  | 868 (87%) | 55 (65%) |  | 923 (85%) |  |
|  | Missing | 23 | 20 |  | 27 | | 16 |  | 27 | 16 |  | 43 |  |
| **Been victim of violence** | Yes | 161 (15%) | 1 (50%) | 0.1630 | 142 (14%) | | 20 (24%) | **0.0170** | 142 (14%) | 20 (24%) | **0.0170** | 162 (15%) |  |
|  | No | 924 (85%) | 1 (50%) |  | 861 (86%) | | 64 (76%) |  | 861 (86%) | 64 (76%) |  | 925 (85%) |  |
|  | Missing | 23 | 20 |  | 27 | | 16 |  | 27 | 16 |  | 43 |  |
| **Been perpetrator of violence** | Yes | 133 (12%) | 1 (50%) | 0.1041 | 114 (11%) | | 20 (24%) | **0.0008** | 114 (11%) | 20 (24%) | **0.0008** | 134 (12%) |  |
|  | No | 955 (88%) | 1 (50%) |  | 892 (89%) | | 64 (76%) |  | 892 (89%) | 64 (76%) |  | 956 (88%) |  |
|  | Missing | 20 | 20 |  | 24 | | 16 |  | 24 | 16 |  | 40 |  |
| **Victim and/or perpetrator of violence** | Yes | 214 (20%) | 1 (50%) | 0.2819 | 187 (19%) | | 28 (33%) | **0.0011** | 187 (19%) | 28 (33%) | **0.0011** | 215 (20%) |  |
|  | No | 873 (80%) | 1 (50%) |  | 818 (81%) | | 56 (67%) |  | 818 (81%) | 56 (67%) |  | 874 (80%) |  |
|  | Missing | 21 | 20 |  | 25 | | 16 |  | 25 | 16 |  | 41 |  |
| **Family try to help me** | Yes | 794 (73%) | 3 (100.0%) | 0.2938 | 734 (73%) | | 63 (75%) | 0.6961 | 734 (73%) | 63 (75%) | 0.6961 | 797 (73%) |  |
|  | No | 292 (27%) | - |  | 271 (27%) | | 21 (25%) |  | 271 (27%) | 21 (25%) |  | 292 (27%) |  |
|  | Missing | 22 | 19 |  | 25 | | 16 |  | 25 | 16 |  | 41 |  |
| **Friends try to help me** | Yes | 675 (62%) | 2 (100.0%) | 0.2712 | 626 (62%) | | 51 (61%) | 0.8613 | 626 (62%) | 51 (61%) | 0.8613 | 677 (62%) |  |
|  | No | 409 (38%) | - |  | 377 (38%) | | 32 (39%) |  | 377 (38%) | 32 (39%) |  | 409 (38%) |  |
|  | Missing | 24 | 20 |  | 27 | | 17 |  | 27 | 17 |  | 44 |  |
| **Physical functioning subscale score** | mean (SD) | 44.07 (9.24) | 43.10 (9.28) | 0.7978 | 44.23 (9.12) | | 42.07 (10.34) | **0.0389** | 44.23 (9.12) | 42.07 (10.34) | **0.0389** | 44.07 (9.23) |  |
|  | median (min, max) | 48.33 (21.46, 54.05) | 40.07 (30.31, 54.05) | 0.7640 | 48.33 (21.46, 54.05) | | 40.07 (21.46, 54.05) | 0.0910 | 48.33 (21.46, 54.05) | 40.07 (21.46, 54.05) | 0.0910 | 48.33 (21.46, 54.05) |  |
| **Role-physical sub-scale score** | mean (SD) | 42.45 (10.02) | 45.17 (7.53) | 0.5072 | 42.57 (9.98) | | 41.20 (10.22) | 0.2264 | 42.57 (9.98) | 41.20 (10.22) | 0.2264 | 42.47 (10.00) |  |
|  | median (min, max) | 46.92 (23.01, 53.98) | 42.82 (38.71, 53.98) | 0.5947 | 46.92 (23.01, 53.98) | | 38.71 (23.01, 53.98) | 0.2303 | 46.92 (23.01, 53.98) | 38.71 (23.01, 53.98) | 0.2303 | 46.92 (23.01, 53.98) |  |
| **Bodily pain subscale score** | mean (SD) | 47.31 (9.97) | 45.60 (8.47) | 0.6747 | 47.49 (9.94) | | 45.01 (9.98) | **0.0279** | 47.49 (9.94) | 45.01 (9.98) | **0.0279** | 47.30 (9.96) |  |
|  | median (min, max) | 47.67 (25.45, 60.77) | 47.67 (31.48, 53.35) | 0.6658 | 47.67 (25.45, 60.77) | | 40.07 (25.45, 60.77) | **0.0204** | 47.67 (25.45, 60.77) | 40.07 (25.45, 60.77) | **0.0204** | 47.67 (25.45, 60.77) |  |
| **General health subscale score** | mean (SD) | 39.92 (8.46) | 36.26 (9.92) | 0.2912 | 39.89 (8.40) | | 40.02 (9.31) | 0.8979 | 39.89 (8.40) | 40.02 (9.31) | 0.8979 | 39.90 (8.46) |  |
|  | median (min, max) | 38.41 (22.81, 59.45) | 35.49 (22.81, 52.83) | 0.2714 | 38.41 (22.81, 59.45) | | 38.41 (22.81, 59.45) | 0.9499 | 38.41 (22.81, 59.45) | 38.41 (22.81, 59.45) | 0.9499 | 38.41 (22.81, 59.45) |  |
| **Vitality subscale score** | mean (SD) | 44.57 (8.98) | 46.57 (10.49) | 0.5867 | 44.59 (8.94) | | 44.41 (9.46) | 0.8585 | 44.59 (8.94) | 44.41 (9.46) | 0.8585 | 44.58 (8.98) |  |
|  | median (min, max) | 45.16 (28.14, 61.83) | 45.16 (35.81, 61.83) | 0.6495 | 45.16 (28.14, 61.83) | | 45.16 (28.14, 61.83) | 0.8245 | 45.16 (28.14, 61.83) | 45.16 (28.14, 61.83) | 0.8245 | 45.16 (28.14, 61.83) |  |
| **Social functioning subscale score** | mean (SD) | 38.90 (10.11) | 37.99 (9.84) | 0.8266 | 38.97 (10.09) | | 37.98 (10.36) | 0.3905 | 38.97 (10.09) | 37.98 (10.36) | 0.3905 | 38.89 (10.11) |  |
|  | median (min, max) | 40.41 (23.44, 55.25) | 34.97 (29.53, 49.47) | 0.8448 | 40.41 (23.44, 55.25) | | 40.41 (23.44, 55.25) | 0.3695 | 40.41 (23.44, 55.25) | 40.41 (23.44, 55.25) | 0.3695 | 40.41 (23.44, 55.25) |  |
| **Role-emotional subscale score** | mean (SD) | 37.89 (9.10) | 37.67 (7.35) | 0.9529 | 37.96 (9.11) | | 37.03 (8.74) | 0.3712 | 37.96 (9.11) | 37.03 (8.74) | 0.3712 | 37.89 (9.08) |  |
|  | median (min, max) | 38.09 (21.66, 52.42) | 38.09 (29.25, 45.66) | 0.9882 | 38.09 (21.66, 52.42) | | 38.09 (21.66, 52.42) | 0.3781 | 38.09 (21.66, 52.42) | 38.09 (21.66, 52.42) | 0.3781 | 38.09 (21.66, 52.42) |  |
| **Mental health subscale score** | mean (SD) | 35.42 (10.77) | 34.87 (8.19) | 0.9001 | 35.37 (10.74) | | 36.02 (11.00) | 0.5949 | 35.37 (10.74) | 36.02 (11.00) | 0.5949 | 35.42 (10.75) |  |
|  | median (min, max) | 31.62 (21.40, 56.79) | 36.58 (21.40, 41.53) | 0.9850 | 31.62 (21.40, 56.79) | | 31.62 (21.40, 56.79) | 0.5952 | 31.62 (21.40, 56.79) | 31.62 (21.40, 56.79) | 0.5952 | 31.62 (21.40, 56.79) |  |
| **Physical component summary score** | mean (SD) | 45.22 (10.44) | 45.25 (11.12) | 0.9946 | 45.39 (10.30) | | 43.12 (11.84) | 0.0576 | 45.39 (10.30) | 43.12 (11.84) | 0.0576 | 45.22 (10.44) |  |
|  | median (min, max) | 47.03 (16.81, 66.45) | 45.28 (32.06, 59.31) | 0.9937 | 47.13 (16.81, 66.45) | | 43.82 (19.12, 65.05) | 0.0998 | 47.13 (16.81, 66.45) | 43.82 (19.12, 65.05) | 0.0998 | 47.03 (16.81, 66.45) |  |
| **Mental component summary score** | mean (SD) | 33.99 (12.12) | 33.51 (12.23) | 0.9219 | 33.97 (12.09) | | 34.23 (12.45) | 0.8533 | 33.97 (12.09) | 34.23 (12.45) | 0.8533 | 33.99 (12.11) |  |
|  | median (min, max) | 32.04 (8.50, 63.87) | 33.75 (17.23, 47.18) | 0.9152 | 32.09 (8.50, 63.87) | | 31.97 (12.49, 60.61) | 0.8353 | 32.09 (8.50, 63.87) | 31.97 (12.49, 60.61) | 0.8353 | 32.04 (8.50, 63.87) |  |

ARIA, Accessibility/Remoteness Index of Australia; AUDIT, Alcohol Use Disorder Identification Test; SEIFA, Socio-Economic Indexes for Areas.
